# Developments in kidney xenotransplantation

**DOI:** 10.3389/fimmu.2023.1242478

**Published:** 2024-01-11

**Authors:** Haiyan Xu, Xiaozhou He

**Affiliations:** Urology Department, Third Affiliated Hospital of Soochow University, Changzhou, China

**Keywords:** kidney xenotransplantation, α-Gal, CD40L-CD40, gene editing, tolerance induction

## Abstract

The search for kidney xenografts that are appropriate for patients with end-stage renal disease has been ongoing since the beginning of the last century. The major cause of xenograft loss is hyperacute and acute rejection, and this has almost been overcome via scientific progress. The success of two pre-clinical trials of α1,3-galactosyltransferase gene-knockout porcine kidneys in brain-dead patients in 2021 triggered research enthusiasm for kidney xenotransplantation. This minireview summarizes key issues from an immunological perspective: the discovery of key xenoantigens, investigations into key co-stimulatory signal inhibition, gene-editing technology, and immune tolerance induction. Further developments in immunology, particularly immunometabolism, might help promote the long-term outcomes of kidney xenografts.

## 1 Introduction

**Figure 1:**
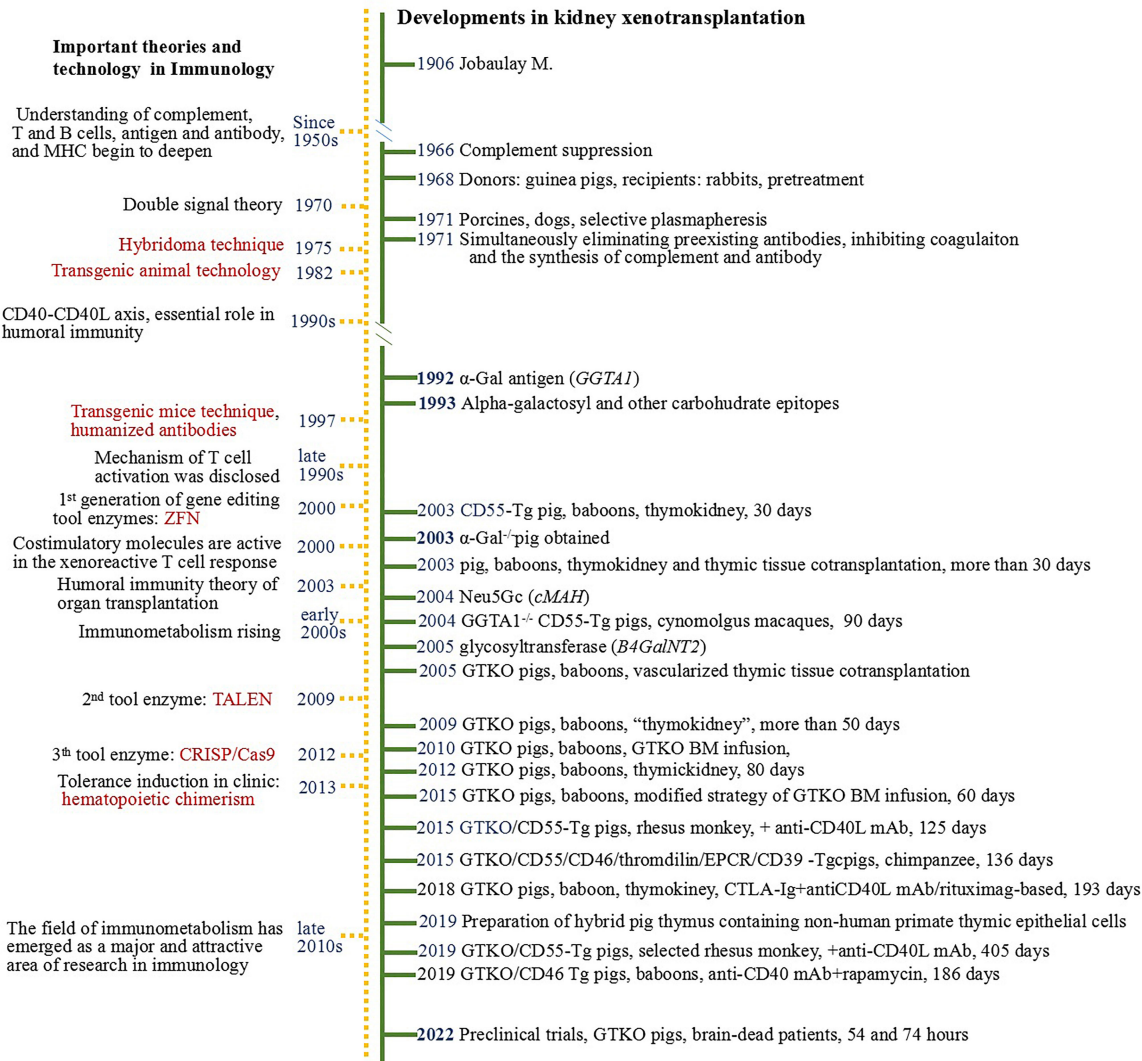
Timeline of developments in kidney xenotransplantation 1906-2022.

Xenotransplantation can play key roles in reducing the kidney donor shortage. Since the first kidney xenotransplant in 1906 (1), great strides have led to achievements in xenotransplantation such that the risk of hyperacute and acute rejection is almost overcome (2, 3). Significant progress has been made in key issues in xenotransplantation (4–6). Important events in kidney xenotransplantation and the advancements of immunological theories and techniques in corresponding periods are listed in **Figure 1**.

Here we especially discuss the pivotal developments of kidney xenotransplantation from an immunological perspective (**Table 1**).

**Table 1:** Critical progress in promoting kidney xenograft survival.

**Discovery of xenoantigens**	
Carbohydrate antigen	α-Gal
	Non-α-Gal
Proteantigen	SLA
**Investigation into key co-stimulatory signal pathways**	
Anti-CD40/anti-CD40L	
**Establishment of genetically engineered pigs**	
CRISPR/Cas9//Human CD55, CD59, CD46, CD39	
**Immune tolerance induction by chimerism**	
“Thymus kidney”	
Bone marrow/Hematopoietic cells	
**Research interests**	

## 2 Key xenoantigens

The recognition of xenoantigens involved in hyperacute rejection has been a long and tortuous road. The first interzygotic twin transplantation in 1953 resulted in long recipient survival and revealed a new direction for organ transplantation. With the discovery and application of immunosuppressive agents, hyperacute rejection after allotransplantation could be controlled, and the survival of recipients gradually increased. However, hyperacute rejection after xenotransplantation cannot be controlled by the empirical application of immunosuppressive agents (7).

Recipient rabbits treated with homogenized guinea pig liver mixtures survived longer after guinea pig kidney grafts were transplanted (8). This inspired many attempts to reduce hyperacute rejection of xenografts, such as the selective removal of plasma components (9), elimination of extant antibodies, inhibition of coagulation, as well as the synthesis of complement and antibodies (10). The results suggested that hyperacute rejection of xenografts is strongly associated with donor antigens, plasma composition, and antibody synthesis, similar to hyperacute rejection during allotransplantation.

### 2.1 α-Gal antigen

The red blood cell surface galactose antigen (DGalα1→3DGal) that induces hyperacute homotransplant rejection due to an ABO mismatch was identified in the late 1980s (11). During xenotransplantation, hyperacute rejection results in an abnormal increase in immunoglobulin (Ig)M serum levels rather than in IgG levels. This indicates that the recipient’s immune system first recognizes the specific antigens harbored in xenografts.

Due to the emergence of monoclonal antibodies (mAbs) using hybridomas, human anti-swine antibodies waere generated and used to identify significant carbohydrate structures for xenotransplantation (12). Then the α-galactosyltransferase (α-Gal) was found, which is encoded by the α-1,3-galactosyltransferase (*GGTA1*) gene (13). Other carbohydrate antigens, such as non-fucosylated chondroitin sulfate monolayers and linear antigens, are also found, locating on the surfaces of all porcine vascular endothelial cells. These antigens tightly bind to anti-Gal isogalectin β4 antibodies and specifically bind to natural, human anti-α-Gal antibodies. Gal epitopes are expressed abundantly in the brush margins of proximal convoluted tubules, moderately in distal convoluted tubules, and not at all in renal collecting tubules and glomeruli. A specific antigen-antibody reaction activates the complement system, leading to a powerful cytotoxic effect that leads to hyperacute grafts (14–18). The discovery of the α-Gal antigen was a major breakthrough in xenotransplantation.

Thereafter, considerable efforts were directed toward decreasing hyperacute rejection of kidney xenotransplants by removing anti-porcine antibodies *in vitro*, short-term infusions of specific carbohydrates (19), or the absorption of anti-xenoantigen antibodies produced in the spleen and kidneys (20). Soluble Gal proteins can partially inhibit human rejection of porcine kidneys. Intravenous infusions of bovine serum albumin-Gal *in vivo* can essentially maintain the depletion of circulating anti-Gal antibodies and prevent or delay antibody deposition and the acute humoral rejection of pig-to-baboon xenografts, but it might be associated with liver damage (21).

### 2.2 Non-α-galantigens (Neu5Gc, CMAH and B4GalNT2)

Transgenic technology was established in 1981 using microinjections; and a transgenic mouse model was created in 1982. The first generation of the gene-editing tool, zinc finger nuclease, was introduced during the late 1990s, and another, transcriptional activator-like effector nuclease, was identified in 2009. These gene-editing techniques had a positive global impact on life sciences.

Pigs with α-Gal knockout (α-Gal^-/-^, GTKO) are important xenotransplantation models (22–24). In the α-Gal^-/-^ pigs to baboon kidney xenotransplantation models, most recipients did not develop hyperacute rejection; however, they succumbed to acute humoral rejection. The significantly increased abundance of peripheral anti-non-α-Gal antibodies in recipients suggested that non-α-Gal antigens in kidney xenografts might trigger the production of large amounts of corresponding antibodies. Thereafter, non-α-Gal antigens were recognized as obstacles to α-Gal^-/-^ pig organ xenotransplantation (25). The α-Gal antigen is crucial for hyperacute rejection, and non-α-Gal antigens play important roles in humoral rejection of xenotransplants. In addition to α-Gal and non-α-Gal, other carbohydrate antigens have a complex spatial distribution in porcine kidneys and are strongly associated with the outcome of porcine kidney xenotransplantation (26).

Non-α-Gal antigens, such as N-glycolylneuraminic acid (Neu5Gc; HD antigen), encoded by the cytidine monophospho-N-acetylneuraminic acid hydroxylase (*cMAH*) gene have been identified (27, 28). Compared with GGTA1^-/-^ pig xenotransplantation, humoral rejection is reduced in GGTA1^-/-^/CMAH^-/-^ pigs xenotransplantation (29), implying that the immune heritability of the Neu5Gc antigen potentially plays an important role in pig-human xenotransplantation. The other carbohydrate non-α-Gal antigen, glycosyltransferase, (SD(a) antigen), is encoded by the β-1,4-N-acetyl-galactosaminyl transferase (*B4GalNT2*) gene (30, 31).

Clustered regularly interspaced short palindromic repeats (CRISPR)-associated protein (Cas9) is a third-generation gene-editing tool. Porcine embryonic fibroblasts with GGTA1^-/-^/Gal^-/-^ were initially created using CRISPR/Cas9 in 2014 (32). Since then, CRISPR/Cas9 has become the preferred means of generating genetically engineered pigs. The serum of many waitlisted patients contained only a minimal number of antibodies that reacted with peripheral blood mononuclear cells from GGTA1^-/-^/CMAH^-/-^/B4GalNT2^-/-^ pigs. However, anti-human leukocyte antigen antibodies in some sensitized patients cross-reacted with porcine major histocompatibility complex (MHC) I antibodies (33). Pigs with simultaneous MHC and three antigen (GGTA1/CMAH/B4GalNT2) inactivation have been generated using the CRISPR/Cas method (34). Natural and inducible anti-SDa plays important roles in GTKO pig-to-rhesus monkey xenotransplant rejection, thus providing further support for the notion that Gal and SDa antigens should be simultaneously targeted (35). Exploration of new key non-α-Gal antigens is currently underway (36).

### 2.3 SLAs

SLAs are being discovered to play an important role in swine innate and adoptive immune responses. In some sensitized kidney transplant-waitlisted patients, some human leucocyte antigen (HLA) antibodies cross-react with SLA class I (37). SLA II is also a xenoantigen (38, 39). And triple (GGTA1, CMAH, B2M) genes modified pigs expressed the SLA I^low^ phenotype, which effects on immune status and susceptibility to human immune responses (40). *In vitro* human TNF-α could increase SLA I expression, while human IL-17 could decrease TNF-α-mediated SLA-I upregulation (41), and downregulation of SLA expression decreases the strength of xenogeneic immune responses towards renal tubular epithelial cells (42). These data may support the SLA-silencing strategy application to prevent xenogeneic cellular immune responses.

## 3 Blocking CD40L-CD40 co-stimulatory signals

Diversity and specificity of immunoglobulins suggests that cellular and humoral immune responses are not separate entities, but complementary components. T and B lymphocytes interact to activate and differentiate into effector cells under specific circumstances. During this process, co-stimulatory signals, such as cluster of differentiation (CD) 40 and its ligand CD40L, CD28-B7, and inducible T cell co-stimulator ligand (ICOS) and its ligand ICOSL, play indispensable roles, and the effects of CD40L-CD40 signaling on xenotransplantation have been extensively investigated.

The 35 kDa polypeptide CD40 is mainly expressed in B lymphocytes (43, 44). After CD40L was identified (44–46), numerous *in vivo* and *in vitro* findings showed that the CD40L-CD40 pathway is essential for T cell responses and specific antibody production by B lymphocytes (47–52). The biological effects of anti-CD40L mAb, as well as other related mAbs, including anti-CD80, anti-CD86 mAbs, and biologicals, such as hCTLA4-Ig, have been extensively studied *in vitro* and *in vivo* (53–55). Results suggest that blocking the CD40-CD40L pathway, or combined blocking of the CD28-B7 signal could effectively inhibit T cell activation and suppress the production of specific antibodies.

Data from pig to non-human primates (NHPs) organ xenotransplants reveal that anti-CD40L mAb suppresses CD40-CD40L co-stimulatory signals and decreases T cell-mediated immune responses, whereas natural anti-Gal antibodies are detectable at baseline (56). The application of anti-CD40L mAbs to NHPs is safe (57, 58) and blocking the CD40L-CD40 signal might induce immune nonresponse to a xenotransplant (59, 60); thus, prolonging xenograft survival (61–64). By comparison, co-stimulation blockades with an anti-CD40L agent is more successful than with an anti-CD40 agent (65–67).

Currently, the immunosuppressive regimen based on the blockade of the CD40-CD40L co-stimulation pathway is considered as an extremely important development in the xenotransplantation. As a biological agent, the affinity and effective doses of these mAbs for individuals, the mechanism of action, and the potential side effects, require further investigation.

## 4 Genetically engineered pig establishment

Expression of the end-stage complement suppressor human CD59 seems to promote the survival of transplanted organs *in vitro* (68, 69). The complement protein CD55 (decay acceleration factor) regulates complements, whereas CD46 is an inhibitory regulator of the complement system. Knocking human CD55, CD59, and CD46 into the pig genomes resulted in their expression in vascular endothelial cells and suppressed damage caused by complement activation (70). Cynomolgus monkeys that received GGTA1^-/-^*/*CD55 transgene (Tg) pig kidneys survived for >90 days (71), which was surprising at the time. This also suggested that human CD55 knock-in promotes xenograft survival, in addition to preventing ureteral stenosis. Recipient rhesus monkeys with low levels of anti-pig antibodies were screened as recipients of GTKO/human CD55 Tg pigs’ kidneys, and the anti-CD40L mAbs applied after transplantation and conventional immunosuppressive protocol resulted in the recipients surviving for >125 days (72).

Thrombomodulin, endothelial protein C receptors, CD39, and other factors function in the regulation of human coagulation. Thrombomodulin and CD39 are involved in complement activation and the coagulation cascade during heterogeneous immune regulation (73–75). In the GTKO/human CD46, CD55, thrombomodulin, endothelial protein C receptors, and CD39 Tg porcine to baboon kidney xenotransplantation models, recipients who received anti-thymocyte globulin (ATG) and anti-CD20 mAb induction, along with anti-CD40 mAb-based immunosuppression therapy survived for up to 136 days (76). In the GTKO/human CD55 Tg porcine to rhesus monkey kidney xenotransplantation models, rhesus monkeys with low antibody titers were selected, some who received transient pan-T cell destruction and the anti-CD40L mAb-based immunotherapy protocol survived for 405 days (77).

The obtained experience in kidney xenotransplantation of genetically engineered pigs to NHPs has provided a solid foundation for pre-clinical trials. The surgeries, α-Gal knockout pigs to brain-dead patient kidney xenotransplantation, were conducted in the USA in 2021, and the survival of xenografts was 54 (2) and 74 (3) h.

## 5 Tolerance induction by chimerism

### 5.1 Thymus co-transplantation

Attempts to induce immune tolerance in xenografts by multiple low-dose xenoantigen inoculations have been unsuccessful. Transplanting fetal porcine thymus and liver tissues into mice to eliminate T and natural killer cells and removing the thymus induces specific tolerance to porcine antigens (78). The mouse CD4^+^ T cell repertoire developed in implanted pig thymus grafts indicated positive selection by porcine (xenogeneic) MHC antigens and negative selection by both mice (recipients) and porcine MHC; this suggested a high level of tolerant immunocompetence (79–81). Findings of kidney allotransplantation in large animals have indicated that the thymus is essential for rapid and stable immune tolerance (82, 83), implying the potential value of thymus transplants to induce tolerance.

The “thymus kidney” was invented by placing thymus tissues under a kidney quilt to facilitate autologous thymus transplantation. The results suggested that the abundance of peripheral CD4^+^CD45RA^+^ T cells increased steadily from 30 to 150 days after transplanting “thymus kidneys” into athymic micropigs, and recipient pigs had acquired immune tolerance. Vascularized donor thymus tissue can induce rapid and stable immune tolerance in recipients to MHC-unmatched allograft (84–86).

In “thymus kidneys” xenotransplantation models, recipient baboons transplanted with a “thymus kidney” graft from a human CD55 Tg pig survived for 30 days, and live thymic epithelial cells and thymic bodies, including a few baboon lymphocytes, were discovered under the renal capsule and omentum of the baboons. The “thymus kidney” can induce the production of non-responsive donor-specific cells and stable amounts of anti-α-Gal antibodies, thus inducing immune tolerance across the genetic immune barrier (87). Transplanting GTKO pig kidneys with the vascular thymus into baboons significantly extended recipients’ survival (88). Recipient baboons with or without cortisol transplanted with “thymus kidneys” from GTKO micropigs survived for >80 days with no signs of cellular rejection or IgG deposition in the transplants and no loss of the transplanted kidneys, suggesting establishment of donor-specific T cell tolerance (89).

Fetal porcine thymus grafts containing mice thymic epithelial cells implanted into mice improved the development of T cells in the thymus, increased the likelihood that they would develop tolerance to the grafts, and reconstructed the T cell population (90). The method for preparing donor thymus grafts enriched with recipient thymic epithelial cells in large animals (cynomolgus monkeys and micropigs) was established (91). This should induce the tolerance of transplanted solid organs, including the kidneys (92).

Mouse T cell receptor–transgenic T cells can be functionally educated using porcine MHC antigens (93). Human T cells develop normally in porcine thymus grafts and form specific tolerance to porcine MHC in immunodeficient mice (94). However, a mouse with a transplanted porcine thymus would develop analogous autoimmune diseases, in which mouse CD4^+^ T cells play a key role (95). Therefore, the differentiation of host T precursor cells in the porcine thymus should differ from the normal physiological state. The number of Tregs in the athymic mice that were grafted with porcine thymus was close to normal, but the regulatory function was not (96). Moreover, T cell differentiation in humanized mice after bone marrow (BM) transplantation revealed that the positive selection was inadequate (97).

These findings should be helpful for thymus transplantation in large animals. Autologous thymus tissues were co-transplanted with GTKO porcine kidneys in the clinical trial of transplantation in two brain-dead patients (2). The results exceeded expectations; however, the mechanisms of tolerance induction need to be further explored.

### 5.2 BM or hematopoietic cell co-transplantation

Transplanted BM or hematopoietic cells can establish chimera-induced tolerance (98). Long-term survival has been achieved using kidneys co-transplanted with BM (99). Moreover, the role of CD4^+^CD25^+^FoxP3^+^Treg cells in these results cannot be ignored (100–102).

Simon et al. (103) injected large doses of porcine spleen cells into baboons and found that low-level chimera status was maintained for almost 1.5 years, during which the baboons did not get sick. These results suggested that donor leukocyte infusion can be used to induce peripheral tolerance during xenotransplantation. Perhaps infusing BM cells with differentiation potential would be more advantageous for establishing chimera-induced immune tolerance.

Griesemer et al. found that baboons transplanted with GTKO BM alone *in vivo* developed peripheral chimeras within 28 days, and the abundance of anti-GTKO porcine antibody or porcine-specific cytotoxicity did not increase. However, anti-porcine and other specific antibodies appeared 14 days after transplantation in baboons that were co-transplanted with BM cells and kidneys, and relatively high levels of anti-Gal antibodies were detected when the porcine kidney was rejected (104). These data suggested that BM infusion is associated with a loss of anti-Gal antibodies. To improve chimerism, the infusion method was modified, and the results were successful, the donor pig kidneys in the two groups survived for 47 and 60 days, respectively (105).

The cell- and species-specific CD47/Signal regulatory protein α (Sirp-α) signaling pathway might be involved in clearing cells derived from porcine BM cells in recipients. Porcine BM transferred the human CD47 gene survived much longer in a recipient baboon, and the chimeras prolonged the survival of porcine skin grafts (106).

## 6 Research interests

In the past decades, many solutions have been applied to solve the ethics and theoretical issues in kidney xenotransplantation, and the breakthrough achieved are encouraging. In addition to immunology-related issues, the transmission of porcine xenotransplantation-relevant viruses (such as porcine endogenous retroviruses, PERV) were well controlled (107). However, whether PERV remains inactivated depends on the stability of porcine genomes after modified by CRISPR/Cas 9 technique.

Comprehensive analysis suggested that, these following issues should be studied in deepth for a better survival of kidney xenografts.

### 6.1 Gene-editing techniques should be perfected

Although CRISPR/Cas9 technology is widely applied, it has some limitations, such as off-target effects, low delivery efficiency, and the immune heritability of Cas9 protein. Any unexpected changes in the human (or xenograft) genome could result in serious and unintended consequences, including the activation of proto-oncogenes and production of new single nucleotide polymorphisms that can alter cellular behavior. In addition, >60% of the population harbors components of humoral and cellular immune responses to Cas9. Therefore, if sustained, Cas9 expression is required during treatment and the immune response induced by the Cas9 must be considered (108). Improvements in CRISPR/Cas9 technology will be conducive to the long-term outcome of clinical kidney xenotransplantation (109).

### 6.2 Deeply investigate the rejection mechanisms of xenotransplantation

#### 6.2.1 Porcine carbohydrate antigens

NHPs often serve as transplant recipients to determine the efficacy of xenotransplantation. However, the expression profile of α-Gal in NHPs differs from that in humans (110). Therefore, data from NHPs can only provide a reference for clinical xenotransplantation. Techniques have been developed to knock out multiple porcine genes (33, 111). However, recent data indicated that the loss of the non-Gal antigen, Neu5Gc, is associated with increased humoral rejection in pig-baboon kidney xenotransplants (112, 113). Therefore, an in-depth investigation of porcine carbohydrate antigens might provide a more comprehensive understanding of their roles in xenotransplantation.

#### 6.2.2 The function and mechanism of novel molecules

In the most recent GTKO pig-baboon kidney xenotransplantation with an anti-CD40 mAb-based immunosuppressive regimen, results indicated that ATG and anti-CD20 mAb eliminated peripheral T and B lymphocytes and inhibited lymphocyte recovery; a decreased abundance of memory CD8^+^ T cells might determine long-term outcomes (114). The hCD47 expression in porcine endothelial cells and podocytes reduced the phagocytic effects of human and baboon macrophages on porcine endothelial cells and podocytes by rectifying the inter-species incompatibility of CD47/Sirp-α signaling (115). Results suggest that the expression of human CD47 in donor pig renal glomerular cells might be an important strategy for preventing proteinuria after xenotransplantation. The results of an *in vivo* study suggested that porcine podocytes expressing hCD47 inhibit the development of albuminuria in GTKO/hCD47 Tg pig-baboon kidney xenotransplantation (116). The underlying mechanism deserves more intensive investigation.

#### 6.2.3 Each type of immune cell involving xenograft rejection

In addition to T and B lymphocyte, monocyte, macrophages, neutrophils, and natural killer (NK) cells should all involve in the initiation and advancements of rejection and outcome of xenografts. Nevertheless, we are just scratching the surface of the iceberg about the function and mechanisms of each type of cells. For instance, NK cells may play an effector role by releasing cytotoxicity granules against xenogeneic cells, or an affector role on other immune cells through cytokine secretion (117), and much work need to be carried out to promote xenograft acceptance by driving NK cells (118).

#### 6.2.4 The discrepancy in metabolism between kidney xenograft donors and human

Pigs, NHPs, and humans significantly differ biologically and physiologically (119–121). All findings suggested that specific immune tolerance induction or immunosuppression regimen need to be developed, and immune mechanism of chronic rejection needs to be explored from multi-angle exploration.

Accumulating evidence suggests that various metabolites and metabolic networks intersect with the induction, regulation, and maintenance of trained immunity (122). Metabolism and the immunological state are inextricably linked, and immunometabolism is recognized as a major mechanism that is central to adaptive and innate immune regulation (123). Now, whether, which, and how metabolites are involved in immune regulation of kidney xenografts remains to be determined. Kidney xenografts grow abnormally in hosts like any other xenograft. The threshold for the ratio of transplanted kidney volume to host body weight is 25 cm^2^/kg; beyond this threshold, kidney xenografts become ischemic (124). This phenomenon reflects physiological differences between GTKO pigs and baboons and more importantly, a link between metabolism and the renal xenograft immune response. This is confirmed by the results that rituximab and CTLA4Ig might confer benefits in terms of symptomatic treatments (125–127).

## 7 Conclusion

Compared with the understanding of the alloimmune response, that of the heterologous immune mechanism is still in its infancy (128, 129). We believe that a deeper understanding of immunological theories and the development of techniques will continue to promote the progress of kidney xenotransplantation. Further studies of immunomechanisms in kidney xenotransplantation might help to promote the survival of kidney xenografts.

## Author contributions

XH wrote the manuscript, and HX revised and reviewed the manuscript. All authors were involved in the creation of the manuscript and are responsible for the content of the work. All authors contributed to the article and approved the submitted version.
